# Impact of Clinical Markers of Nutritional Status and Feeding Jejunostomy Use on Outcomes in Esophageal Cancer Patients Undergoing Neoadjuvant Chemoradiotherapy

**DOI:** 10.3390/nu12103177

**Published:** 2020-10-17

**Authors:** Rishi Jain, Talha Shaikh, Jia-Llon Yee, Cherry Au, Crystal S. Denlinger, Elizabeth Handorf, Joshua E. Meyer, Efrat Dotan

**Affiliations:** 1 Fox Chase Cancer Center, Department of Hematology/Oncology, Philadelphia, PA 19111-2497, USA; jiallony@gmail.com (J.-L.Y.); Cherry.au.001@gmail.com (C.A.); crystal.denlinger@fccc.edu (C.S.D.); efrat.dotan@fccc.edu (E.D.); 2 Fox Chase Cancer Center, Department of Radiation Oncology, Philadelphia, PA 19111-2497, USA; talha.s019@gmail.com (T.S.); joshua.meyer@fccc.edu (J.E.M.); 3 Biostatistics, Fox Chase Cancer Center, Philadelphia, PA 19111-2497, USA; elizabeth.handorf@fccc.edu

**Keywords:** nutrition, esophageal cancer, neoadjuvant chemoradiation, enteral nutrition

## Abstract

Background: Patients with esophageal cancer (EC) have high rates of malnutrition due to tumor location and treatment-related toxicity. Various strategies are used to improve nutritional status in patients with EC including oral and enteral support. Methods: We conducted a retrospective analysis to determine the impact of malnutrition and prophylactic feeding jejunostomy tube (FJT) placement on toxicity and outcomes in patients with localized EC who were treated with neoadjuvant chemoradiation therapy (nCRT) followed by esophagectomy. Results: We identified 125 patients who were treated with nCRT between 2002 and 2014. Weight loss and hypoalbuminemia occurred frequently during nCRT and were associated with multiple adverse toxicity outcomes including hematologic toxicity, nonhematologic toxicity, grade ≥3 toxicity, and hospitalizations. After adjusting for relevant covariates including the specific nCRT chemotherapy regimen received and the onset of toxicity, there were no significant associations between hypoalbuminemia, weight loss, or FJT placement and relapse-free survival (RFS) or overall survival (OS). FJT placement was associated with less weight loss during nCRT (*p* = 0.003) but was not associated with reduced toxicity or improved survival. Conclusions: Weight and albumin loss during nCRT for EC are important factors relating to treatment toxicity but not RFS or OS. While pretreatment FJT placement may reduce weight loss, it may not impact treatment tolerance or survival.

## 1. Introduction

In 2020, approximately 18,440 new cases of esophageal cancer (EC) will be diagnosed in the United States and 16,170 will die from this disease [1]. Unfortunately, despite advances in EC therapy, under 20% of patients survive for five years after diagnosis [1]. Up to 80% of EC patients present with pretreatment malnutrition, largely due to mechanical obstruction [2]. Furthermore, EC is associated with a hyperinflammatory, cachectic state marked by weight loss and sarcopenia [3]. The combination of nutrient imbalance and cancer cachexia leads to loss of muscle mass and resultant loss of physical functioning, reduced quality of life, and increased risk of treatment-related toxicity [3].

Curative intent therapy in EC involves neoadjuvant chemoradiotherapy (nCRT) followed by esophagectomy [4]. However, nCRT carries the risk of significant gastrointestinal (GI) toxicities, including any-grade nausea (53%), vomiting (25%), diarrhea (18%), constipation (27%), and esophagitis (19%), among others [5]. Thus, malnutrition can be further aggravated by the toxicity of nCRT leading to treatment delays or interruptions and worse outcomes [6]. While clinicians use changes in weight or serum albumin levels, consensus is lacking on the most reliable indicator of malnutrition. Commonly used strategies to treat malnutrition in EC may include oral nutritional supplements, enteral nutrition with a percutaneous endoscopic gastrostomy (PEG) or feeding jejunostomy tube (FJT), or even total parenteral nutrition (TPN) in severe cases [7]. Many institutions use preventive placement of enteral feeding support before the initiation of nCRT for EC [8].

While data support the use of postoperative enteral support after esophagectomy [9], limited data are available regarding the optimal method of preoperative nutritional support. While one study of oral nutritional intervention showed that a dietitian-delivered, preoperative nutritional intervention was able to reduce weight loss and decrease surgical morbidity [10], other studies using oral-based interventions have failed to show consistent benefits in outcomes such as nCRT tolerance [11]. The utility of preoperative enteral nutrition in EC has recently been questioned [12]. To date, there is no consensus regarding the optimal management of malnutrition in EC during nCRT. Further, there is little data available regarding the impact of malnutrition occurring during nCRT on outcomes. We conducted a single-center retrospective analysis evaluating the associations between markers of nutritional status and FJT placement on the tolerance and outcomes of patients with localized EC treated with nCRT.

## 2. Materials and Methods

### 2.1. Patient Selection and Data Collection

The study was conducted in accordance with the Declaration of Helsinki, and the protocol was approved by the Institutional Review Board (IRB) of Fox Chase Cancer Center (IRB# 15-9020) on 28 August 2014. Following IRB approval, we retrospectively identified patients with localized EC who underwent nCRT followed by esophagectomy between 2002 and 2014. The tumor registry was utilized to identify eligible patients. The following baseline data at the time of EC diagnosis were collected: age, gender, Eastern Cooperative Oncology Group (ECOG) performance status, comorbidities and Charlson comorbidity index (CCI), smoking status, tumor location, tumor histology, and clinical stage at diagnosis. Former smokers were defined as those who quit >1 year before diagnosis. Data regarding chemotherapy regimen used during radiation, total radiation dose, and pathologic stage were collected.

A detailed pre-nCRT and post-nCRT assessment of nutritional status was conducted for the following factors: placement of pretreatment FJT, weight change during nCRT (lbs), and albumin change during nCRT (g/dL). Pre-nCRT weight and albumin levels were within two weeks of initiation of CRT, while post-nCRT weight and albumin were obtained within one week of nCRT completion. For each patient, weight change was divided into the following categories: (1) gain of body weight, (2) loss of <5% of body weight, (3) loss of 5–10% of body weight, or (4) loss of > 10% of body weight. Albumin change was categorized into either (1) decrease of < 0.5 g/dL or (2) a decrease of ≥0.5 g/dL.

A comprehensive review of clinic notes and laboratory values before, during, and immediately after the completion of nCRT was conducted to identify the onset of toxicity. All toxicity endpoints are defined in Table 1. The onset of hematologic or renal toxicity was scored based on grade and severity per the Common Terminology Criteria for Adverse Events (CTCAE version 4.0). The onset of any-grade nonhematologic toxicity during nCRT was also recorded. Nonhematologic toxicities recorded included the onset of the following: mucositis, nausea, vomiting, diarrhea, constipation, anorexia, dehydration, fatigue, esophagitis, dysphagia, or infections. Due to limitations in clinical documentation, the presence of nonhematologic toxicity endpoints was not graded but rather recorded as binary (yes or no). If a hematologic or nonhematologic toxicity occurred multiple times in the same patient, only one event was included for analysis. We also collected data on any nCRT-related hospitalizations, chemotherapy dose interruptions, or dose reductions. All data collection was completed by two reviewers to ensure accuracy.

### 2.2. Statistical Analysis

Univariate analyses (UVA) were used to determine associations between covariates and nutritional outcomes, using Fisher’s exact test to determine significance of relationships. Multivariable regression analyses (MVA) were used to determine associations between nutritional markers and toxicity, adjusting for the following covariates: age, gender, smoking status, pathologic stage, nCRT regimen, ECOG performance status, and comorbidities (CCI). Linear regression analyses were used to model hematologic and nonhematologic toxicity scores, and logistic regression analyses were used to model the presence or absence of dose reductions/interruptions, hospitalizations, and grade ≥3 toxicities. Multivariable Cox proportional hazards regression models were also used to determine associations between nutritional markers and relapse-free survival (RFS) and overall survival (OS) adjusting for the following covariates: onset of any grade ≥3 toxicity during nCRT, the chemotherapy regimen chosen during nCRT, and also the presence of a pathologic complete response after nCRT. All analyses were performed using Stata software (version 12) StataCorp LLC (College Station, TX, USA), and *p*-values < 0.05 were considered statistically significant.

## 3. Results

### 3.1. Baseline Characteristics and Markers of Nutritional Status

A total of 125 patients who underwent nCRT before esophagectomy for EC were identified. Baseline demographics and disease and treatment characteristics are shown in Table 2. The patients had a median age of 63 and were primarily male (84%). The majority of patients had lower esophageal tumors (88%) and adenocarcinoma (86%). The majority of patients had no comorbidities with a CCI of 0 (70%) and had an ECOG performance score of 0 (56%). Clinical stage III EC was most common (50%), while the most common pathologic stage following esophagectomy was stage II (38%). Pathologic complete response was achieved in 32 patients (26%). The most common chemotherapy regimen used was cisplatin/5-FU in 65 patients (52%), while 45 patients (36%) received carboplatin and paclitaxel. The majority of patients (74%) received ≥5000cGy of total radiation.

### 3.2. Markers of Malnutrition and Toxicity during nCRT

Nutritional characteristics before and after nCRT are shown in Table 3. Mean weight loss during nCRT completion was 10 lbs, with the most common category of weight loss being 5–10% occurring in 36% of patients. Mean albumin decrease during nCRT was 0.4 g/dL. There was an even distribution of patients with or without a pretreatment FJT (62 and 63 patients, respectively). Patients with an FJT placed prior to nCRT had a median year of diagnosis of 2011, while those without FJT placement had a median year of diagnosis of 2007. After nCRT completion, mean weight loss was significantly reduced in patients with an FJT vs. those without an FJT (8 lbs vs. 13 lbs, *p* = 0.003). However, no significant difference in mean albumin loss was noted in those with an FJT vs. those without FJT (0.38 g/dL vs. 0.52 g/dL, *p* = 0.15). The prevalence of nCRT-related toxicity is also shown in Table 3. Chemotherapy dose reductions were required in 10% of patients while dose interruptions were required in 22%. In total, 26% of patients had dose interruptions or reductions and were unable to receive all planned chemotherapy. Eighteen percent of patients had unplanned hospitalizations during nCRT for management of nCRT-related toxicity.

Associations between baseline and treatment characteristics and markers of malnutrition are shown in Table S1. Patients treated with nCRT with cisplatin/5-FU experienced higher percent weight loss compared to those treated with carboplatin/paclitaxel (*p* < 0.001). Patients who received carboplatin/paclitaxel were more likely to have an FJT (84% in carboplatin/paclitaxel versus 31% in cisplatin/5-FU, *p* < 0.001). Patients with an ECOG performance score of 1 were more likely to have an FJT than those with a score of 0 (*p* = 0.025). Otherwise, there were no significant associations between markers of malnutrition or FJT placement and age, gender, smoking status, pathologic stage, or comorbidity score.

### 3.3. Association between Albumin, Weight Loss, and Toxicity during CRT

The results of the UVA and MVA are shown in Table 4. Various markers of malnutrition were associated with toxicity. An albumin loss of ≥0.5 during nCRT was significantly associated with the occurrence of nonhematologic toxicity, hematologic toxicity, and any grade ≥3 toxicity in UVA. After adjusting for covariates in the MVA, an albumin loss of ≥0.5 during nCRT was significantly associated with hospitalizations, nonhematologic toxicity, hematologic toxicity, and any grade ≥3 toxicity. When viewing specific nonhematologic toxicities and albumin change, higher rates of mucositis (61% vs. 36%), nausea (79% vs. 63%), vomiting (47% vs. 37%), diarrhea (30% vs. 24%), and anorexia (52% vs. 37%) were noted in those with an albumin loss of ≥ 0.5 versus albumin loss of <0.5. A weight loss of 5–10% and >10% during nCRT was significantly associated with nonhematologic toxicity in UVA and MVA, while a weight loss of >10% was significantly associated with higher grade ≥3 toxicity in the MVA. There were no associations between pretreatment FJT status and toxicity rates in UVA or MVA.

### 3.4. RFS and OS by Albumin, Weight Loss, and FJT Status

The results of the MVA of RFS and OS by nutritional markers or FJT placement status are shown in Table 5. After adjusting for relevant covariates including the onset of grade ≥ 3 toxicity, the specific chemotherapy regimen used during nCRT, and the presence of a complete response to neoadjuvant therapy, there were no significant associations between FJT status, albumin change, or weight loss and RFS or OS.

## 4. Discussion

Pretreatment malnutrition is a common occurrence in patients with localized EC and may be exacerbated by the toxicity of nCRT used as part of standard trimodality therapy (chemoradiation therapy followed by esophagectomy) [4,6]. As malnutrition and associated muscle loss have been strongly associated with multiple adverse treatment outcomes including increased chemotherapy toxicity [13] and reduced survival [14] in other malignancies, deterioration of nutritional status in patients undergoing nCRT for EC may be detrimental and further compromise survival in this deadly disease. While postesophagectomy nutritional interventions such as FJT placement are beneficial [15], there is little data on the impact of changes in nutritional markers during nCRT on outcomes for EC.

We conducted a single-center retrospective analysis of patients with EC who underwent nCRT prior to esophagectomy. The majority of EC patients either developed or had exacerbation of weight loss and hypoalbuminemia during nCRT. Our data are in line with prior studies showing high rates of malnutrition related to nCRT [16]. Higher degrees of hypoalbuminemia or weight loss during nCRT were associated with significantly higher rates of multiple important toxicity measures, including chemotherapy dose modifications and nonhematologic, hematologic, and grade ≥3 toxicity. Associations between impaired treatment tolerance and malnutrition or sarcopenia have previously been identified with a variety of malignancies and cancer therapies [17,18]. While not thoroughly studied, potential hypotheses linking these conditions include acquired pharmacokinetic differences in drug metabolism in patients with low lean muscle mass, variations of fat-free mass and volume of distribution, among others [19]. Patients with profound weight and muscle loss during nCRT may be unable to receive as much chemotherapy due to missed or reduced chemotherapy doses in the setting of toxicity such as renal dysfunction or cytopenias. Notably, nonhematologic toxicities such as mucositis, nausea, and vomiting were more common in patients with more pronounced hypoalbuminemia. These challenging symptoms are mostly attributed to chemotherapy and may also have necessitated dose modifications resulting in inadequate therapy. This warrants further investigation.

Weight loss was also associated with the type of chemotherapy regimen utilized, with higher rates of weight loss in patients treated with cisplatin/5-FU compared to the more contemporary regimen of carboplatin/paclitaxel. This is likely related to higher GI toxicity, particularly nausea and mucositis, which have been associated with cisplatin/5-FU in clinical trials [20]. Malnutrition in patients undergoing nCRT is likely further compounded by toxicity associated with radiotherapy, especially esophagitis and dysphagia. Some data suggest that neoadjuvant chemotherapy prior to nCRT may reduce malnutrition due to less dysphagia, resultant weight gain, and improved quality of life [21]. More data regarding this approach and its impact on nutritional status and other outcomes are necessary.

Beyond nutritional consultation and use of oral nutritional supplements, enteral support with prophylactic FJT placement prior to nCRT is increasingly common at many centers. In our cohort, FJT placement was most common in patients treated with carboplatin and paclitaxel, reflecting changes in practice patterns. FJT placement was also more common in patients with a higher ECOG performance score, suggesting that reduced baseline functioning may have contributed to the decision for FJT placement. Although performance status is not typically a criterion for FJT use, physicians may choose more aggressive nutritional support mechanisms in those who are weaker or deconditioned. Despite a small reduction in weight loss (5 lbs), there was no reduction in toxicity rates in patients who had an FJT placed prior to nCRT. Other studies have also questioned the utility for preoperative enteral feeding. Studies have shown high rates of tube-related complications without a substantial improvement in nutritional parameters [12,22]. With complication rates up to 40% related to FJT use, including some even requiring operative interventions [23], a detailed risk/benefit discussion must take place with the patient prior to FJT placement. It is likely that certain select patients with critical malnutrition and severe obstruction may benefit most from PEG or FJT placement before nCRT. Given the lack of high-level evidence of enteral support during nCRT, consensus guidelines from major organizations do not mandate enteral support but recommend consideration of FJT placement in those with significant impairment of oral intake [24,25]. Additional prospective research regarding the utility and optimal timing of FJT placement is warranted.

There is little available data regarding changes in nutritional markers during nCRT and survival outcomes. To further understand these relationships in our cohort, we conducted an MVA of nutritional status and RFS/OS after adjusting for relevant covariates including the onset of grade ≥3 toxicity, the specific chemotherapy regimen used during nCRT, and the presence of a complete pathologic response to nCRT, a known prognostic marker. Despite the strong associations between weight loss and albumin loss and key toxicity endpoints in our UVA and MVA, there were no significant associations between weight loss and hypoalbuminemia and RFS/OS. Furthermore, there was no impact of FJT status on survival outcomes. While short-term changes in nutritional status during the 5–6 weeks of nCRT may not impact survival outcomes, additional data are needed to understand the effect of longer-term changes in nutritional markers on survival outcomes. Efforts should be made to identify better biomarkers of nutritional status which may be more reflective of the true burden of malnutrition and the resultant catabolic physiologic state (e.g., markers of inflammation) in this population.

There are limitations of our study. As a single-institution analysis, additional data from other centers would help us understand the impact of nutritional status on toxicity and outcomes in larger populations. The retrospective nature of our study makes it challenging to determine causality of malnutrition with adverse outcomes as other patient, tumor, or treatment-related factors may have varying degrees of contribution. Retrospective evaluation of nutritional status and treatment-related toxicity is challenging given limitations in clinical documentation. To overcome these challenges, each chart was reviewed independently by two reviewers to assess accuracy. Finally, the diagnosis of esophageal cancer in our cohort ranged over a period of 12 years, a time frame when the standard of care for nCRT for localized esophageal cancer evolved as did nutritional support methods and supportive care capabilities. Despite these limitations, given the high morbidity of nCRT and poor survival of patients with esophageal cancer, these preliminary data support ongoing efforts to improve nutritional status during nCRT in an effort to reduce toxicity and improve outcomes.

In the future, assessment of muscle status in conjunction with markers of malnutrition may be useful in selecting patients who would benefit the most from aggressive nutritional support. In those with evidence of severe muscle depletion and systemic inflammation, addition of one of the many anticachexia agents in development in conjunction with nutritional support may be especially advantageous [26]. In cases of severe malnutrition from esophageal tumor obstruction, esophageal stent placement is increasingly used in the palliative setting and can lead to an immediate improvement of obstructive symptoms. While this may prevent the need for enteral nutrition, risks including esophageal fistula formation have been reported when stents were placed prior to chemoradiotherapy [27]. While these strategies hold promise, further prospective evaluation is necessary before standard use in clinical settings.

In this study, we demonstrate strong associations between malnutrition and increased toxicity but not survival among patients with localized EC treated with nCRT. There was no improvement in outcomes with FJT use, which calls into question the utility of prophylactic FJT placement prior to nCRT. Our findings emphasize the need for future research to identify novel strategies to reduce malnutrition and improve the tolerance of nCRT. Future studies will require multidisciplinary strategies—potentially incorporating nutritional support, physical activity, and novel anti-inflammatory/anticachexia pharmacotherapies—with the goal of improved treatment tolerance, quality of life, and, ultimately, survival.

## Figures and Tables

**Table 1:** Definitions of toxicity outcomes.

Hospitalizations	Any Unexpected Hospitalization during nCRT
Nonhematologic toxicity ^1^	Any-grade mucositis, nausea, vomiting, diarrhea, constipation, anorexia, dehydration, fatigue, esophagitis, dysphagia, neurotoxicity
Hematologic toxicity ^2^	Neutropenia, thrombocytopenia, anemia
Grade ≥3 toxicity ^3^	Onset of any grade ≥3 toxicity during nCRT Hgb < 8 g/dL, ANC < 1000/mm3, Plts < 50,000/mm3, Cr < 3× baseline or >3.0 mg/dL
Dose reduction or interruption ^4^	Unplanned chemotherapy dose reduction or interruption

nCRT, neoadjuvant chemoradiation therapy; Hgb, hemoglobin; ANC, absolute neutrophil count; Plts, platelets; Cr, creatinine. ^1^ Includes any-grade nonhematologic toxicity. ^2^ Weighted scores given to higher grade hematologic toxicity. ^3^ Grade ≥3 toxicity including hematologic or renal insufficiency. ^4^ Any dose reduction or interruption regardless of percent of reduction or timing of interruption.

**Table 2:** Patient tumor and treatment characteristics.

Characteristic	Total *N* = 125 (%)
Median Age	63, range 35–80
Gender	
Male	105 (84)
Female	20 (16)
Pathologic subtype	
Adenocarcinoma	108 (86)
Squamous	17 (14)
Location	
Lower tumor	110 (88)
Other	15 (12)
Smoking status	
Past	71 (57)
Current	21 (17)
Former	33 (26)
Charlson comorbidity index	
CCI = 0	87 (70)
CCI = 1	25 (20)
CCI >1	13 (11)
Performance status	
0	70 (56)
≥1 or greater	55 (44)
Clinical stage	
I	1 (0)
II	48 (38)
III	63 (50)
IV ^1^	13 (10)
Pathologic stage	
0 ^2^	32 (26)
I	15 (12)
II	47 (38)
III	24 (19)
IV ^3^	7 (6)
Chemotherapy regimen	
Carboplatin/Paclitaxel	45 (36)
Cisplatin/5-FU	65 (52)
Other ^4^	15 (12)
Radiation dose	
<5000 cGy	33 (26)
≥5000 cGy	92 (74)

CCI, Charlson comorbidity index;. ^1^ Patients with clinical stage IV disease most often had locally advanced lymphadenopathy that was able to be covered by radiation field. ^2^ Pathologic stage was determined after esophagectomy, pathologic stage 0 indicates a pathologic complete response. ^3^ Five were clinical stage IV, two were upstaged at time of surgery. ^4^ Nine treated on clinical trial with combination of Vandetanib, Paclitaxel, Carboplatin, 5-FU.

**Table 3:** Changes in nutritional markers and onset of toxicity during neoadjuvant chemoradiation therapy.

Characteristic	Total *N* = 125 (%)
Mean weight pre-nCRT (lbs)	188
Mean weight post-nCRT	178
Mean weight change	10
Weight change category	
Weight gain	20 (16)
Weight loss <5%	40 (32)
Weight loss ≥5–10%	45 (36)
Weight loss ≥10%	20 (16)
Mean albumin ^1^ (pre-nCRT) (g/dL)	3.8
Mean albumin (post-nCRT) (g/dL)	3.4
Mean albumin (g/dL) loss	0.4
Albumin change	
<0.5 g/dL	59 (47)
≥0.5 g/dL	61 (49)
Pre-CRT feeding jejunostomy tube placement	
Yes	62 (50)
No	63 (50)
Mean weight loss with FJT (lbs)	8
Mean weight loss without FJT (lbs)	13
Mean albumin loss with FJT (g/dL)	0.38
Mean albumin loss without FJT (g/dL)	0.52
Chemotherapy dose reductions	12 (10)
Chemotherapy dose interruptions	27 (22)
Dose reductions/interruptions	33 (26)
Hospitalizations	23 (18)

nCRT, neoadjuvant chemoradiation therapy; FJT, feeding jejunostomy tube; ^1^ Albumin levels were unavailable in 4 patients.

**Table 4:** Associations between nutritional markers and toxicity.

	Albumin Change (<0.5 vs. ≥0.5)		Weight Loss (Gain or <5%, 5–10%, >10%)		J-Tube (Yes vs. No)	
Toxicity	UVA (*p*-value)	MVA (*p* > z or *p* > t)	UVA (*p*-value)	MVA (*p* > z or *p* > t)	UVA (*p*-value)	MVA (*p* > z or *p* > t)
Dose reductions/ interruptions	NS	0.043	NS	NS	NS	NS
Hospitalizations	NS	NS	NS	NS	NS	NS
Nonhematologic toxicity	0.011	0.004	0.019	<5% = NS 5–10% = 0.035 >10% = 0.002	NS	NS
Hematologic toxicity	0.004	0.002	NS	NS	NS	NS
Grade ≥3 toxicity	0.006	0.004	NS	<5% = NS 5–10% = NS >10% = 0.032	NS	NS

UVA, univariate analysis; MVA, multivariable regression analyses; NS, nonsignificant.

**Table 5:** Associations between nutritional markers and relapse-free and overall survival.

	Albumin Change (<0.5 vs. ≥0.5)	Weight Loss (<5% vs. ≥5%)	J-Tube (Yes vs. No)
	MVA HR [95% CI], *p-*Value	MVA HR [95% CI], *p-*Value	MVA HR [95% CI], *p-*Value
Relapse-free survival	1.13 [0.64–2.01], *p* = 0.67	1.29 [0.71–2.32], *p* = 0.40	1.36 [0.69–2.69], *p* = 0.37
Overall survival	0.98 [0.57–1.70], *p* = 0.95	1.27 [0.73–2.24], *p* = 0.40	0.86 [0.47–1.59], *p* = 0.63

MVA, multivariable regression analyses; HR, hazard ratio; CI, confidence interval.

## Supplementary Materials

The following are available online at https://www.mdpi.com/2072-6643/12/10/3177/s1, Table S1: Associations between patient characteristics and markers of nutritional status.
