# Supplementary material for: Impact of Clinical Markers of Nutritional Status and Feeding Jejunostomy Use on Outcomes in Esophageal Cancer Patients Undergoing Neoadjuvant Chemoradiotherapy
Source: Nutrients. 2020 Oct 17;12(10):3177. doi: 10.3390/nu12103177 (PMC7602938; doi:10.3390/nu12103177)
Supplement: Supplementary file 1 [file nutrients-12-03177-s001.pdf]

Table S1. Associations between patient characteristics and markers of nutritional status.

	Albumin change (% of cohort)			Weight loss (% of cohort)					J-tube (% of cohort)		
**Covariates**	**< 0.5**	**≥ 0.5**	***p*-value**	**Gain**	**< 5%**	**5–10%**	**≥ 10%**	***p*-value**	**Yes**	**No**	***p*-value**
Age < 65	46%	54%	NS	10%	36%	34%	19%	NS	48%	52%	NS
Age ≥ 65	48%	52%		26%	24%	38%	12%		52%	48%	
Female	40%	60%	NS	20%	40%	25%	15%	NS	55%	45%	NS
Male	49%	51%		17%	29%	38%	16%		49%	51%	
Never smoker	36%	64%	NS	15%	36%	39%	9%	NS	55%	45%	NS
Past smoker	51%	49%		20%	28%	34%	18%		45%	55%	
Active smoker	52%	48%		14%	29%	38%	19%		57%	43%	
Path stage 0	53%	47%	NS	13%	34%	44%	9%	NS	38%	63%	NS
Path stage 1	67%	33%		33%	27%	27%	13%		53%	47%	
Path stage 2	45%	55%		23%	23%	32%	21%		53%	47%	
Path stage 3	38%	63%		8%	33%	46%	13%		54%	46%	
Path stage 4	29%	71%		0%	57%	14%	29%		57%	43%	
Carbo/Paclitaxel	51%	49%	NS	38%	33%	27%	2%	<0.001	84%	16%	<0.001
Cisplatin/5-FU	43%	57%		8%	26%	42%	25%		31%	69%	
Other	53%	47%		0%	40%	40%	20%		27%	73%	
PS 0	47%	53%	NS	20%	27%	41%	12%	NS	41%	59%	0.025
PS 1	50%	50%		15%	37%	29%	19%		63%	37%	
PS ≥ 2 [1]	33%	67%		0%	0%	67%	33%		33%	67%	
CCI 0-1	48%	51%	NS	16%	32%	34%	17%	NS	50%	50%	NS
CCI >1	54%	46%		31%	15%	46%	8%		46%	54%	

NS, nonsignificant; PS, performance status; CCI, Charlson comorbidity index. [1] Only 4 patients had a PS 2 or greater.
